# Supplementary figures and images for: Adaptive Protein Evolution in Animals and the Effective Population Size Hypothesis
Source: PLoS Genet. 2016 Jan 11;12(1):e1005774. doi: 10.1371/journal.pgen.1005774 (PMC4709115; doi:10.1371/journal.pgen.1005774)

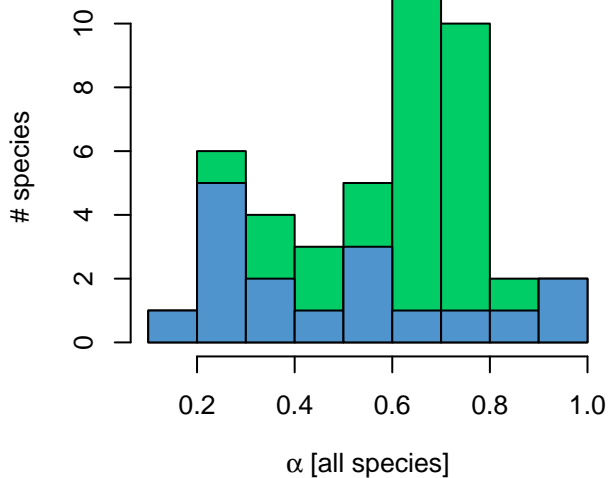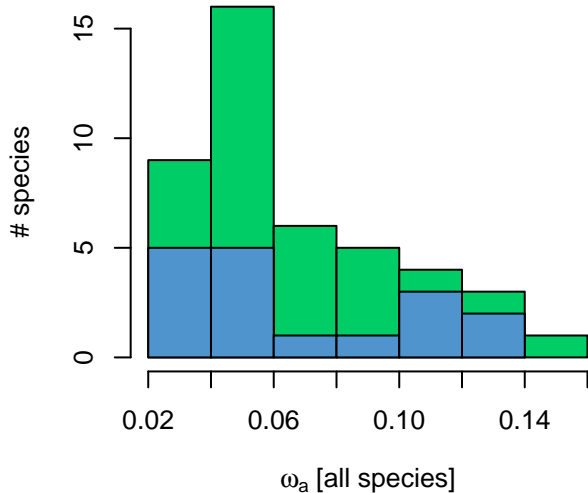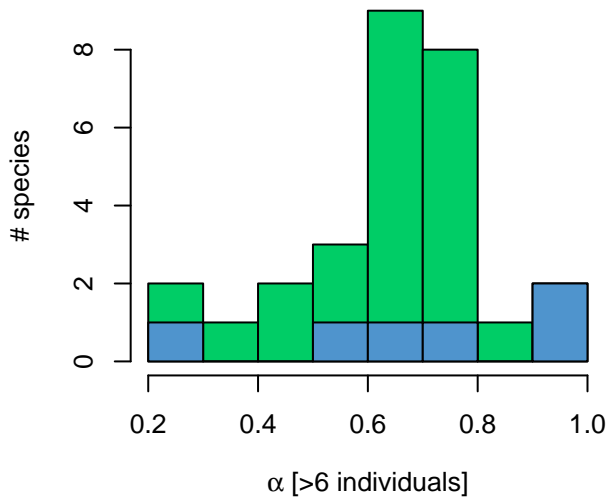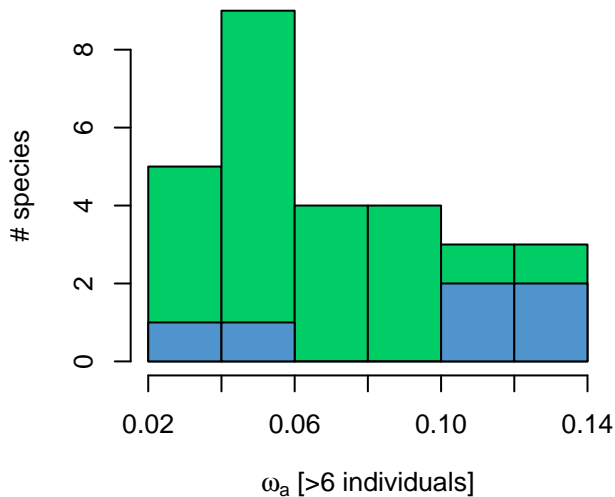

Supplement: S1 Fig — Top: all species; Bottom: 26 species for which at least seven individuals have been sampled. (PDF) [file pgen.1005774.s002.pdf]

a

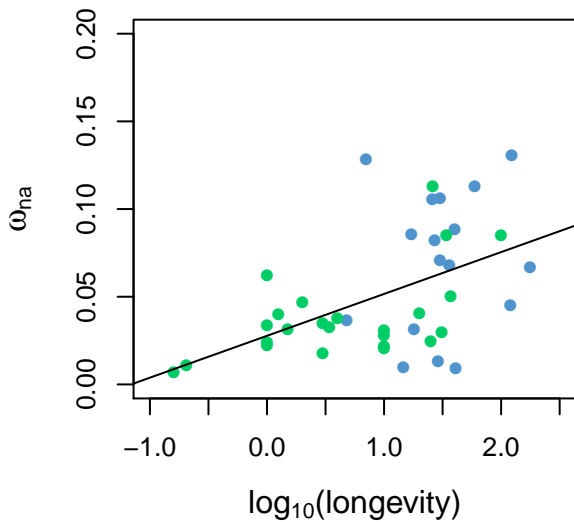

b

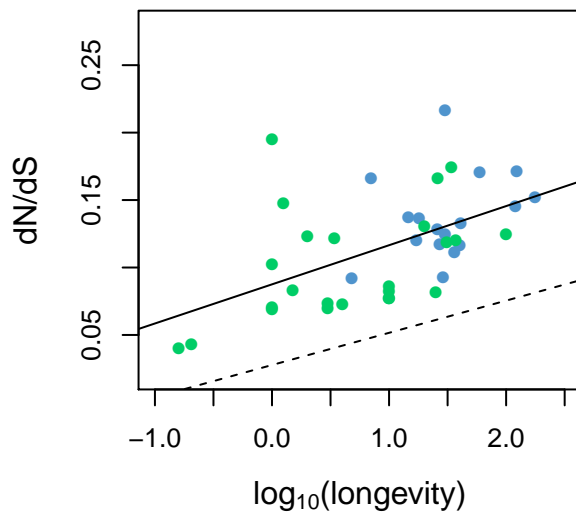

c

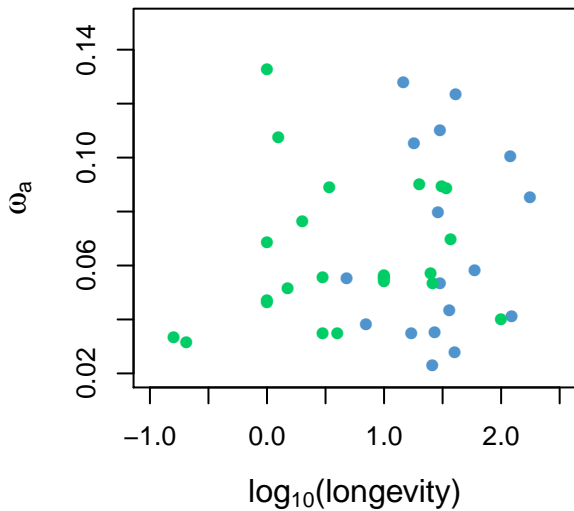

d

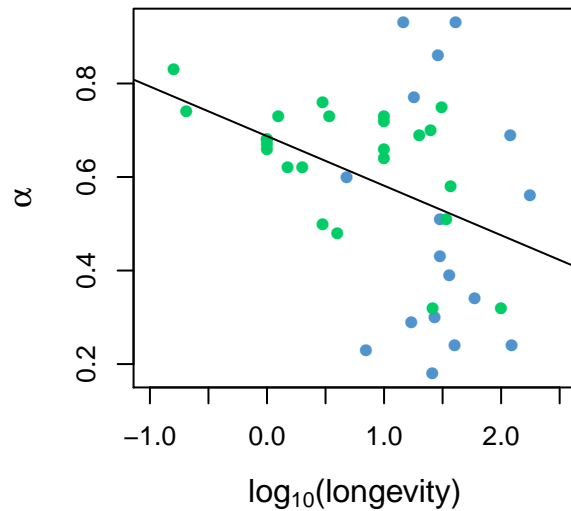

Supplement: S2 Fig — n = 41 species; blue: vertebrates; green: invertebrates; a: r2 = 0.25, p-val<10−3; b: r2 = 0.28, p-val<10−3; c: r2 = 0.02, not significant; d: r2 = 0.15, p-val = 0.01. (PDF) [file pgen.1005774.s003.pdf]

a

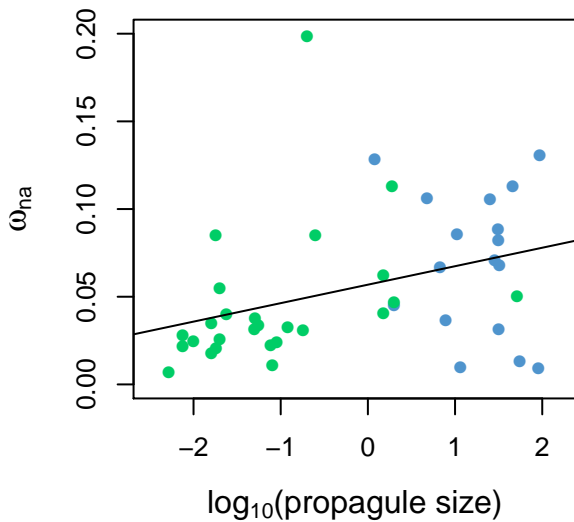

b

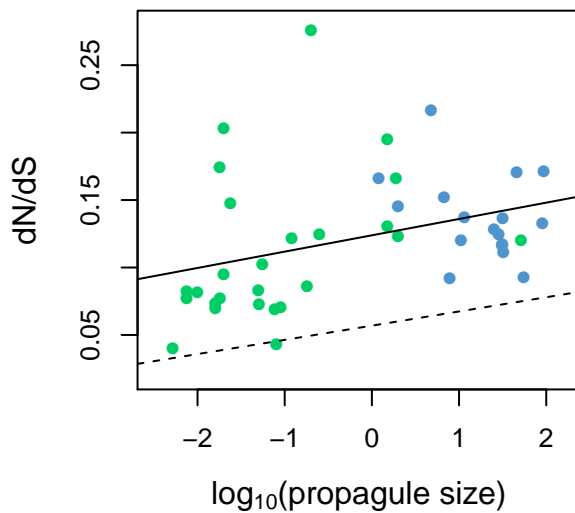

c

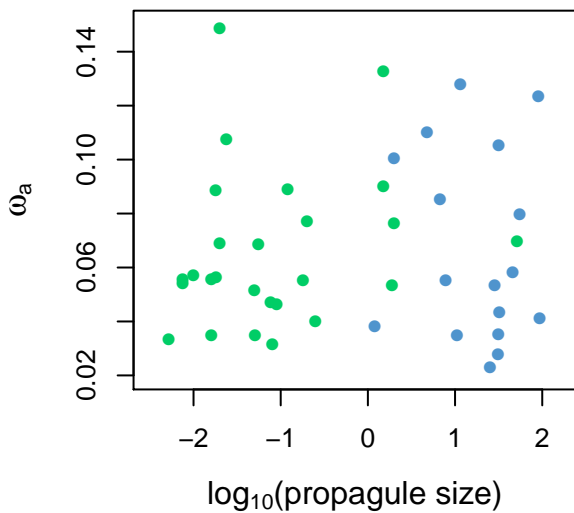

d

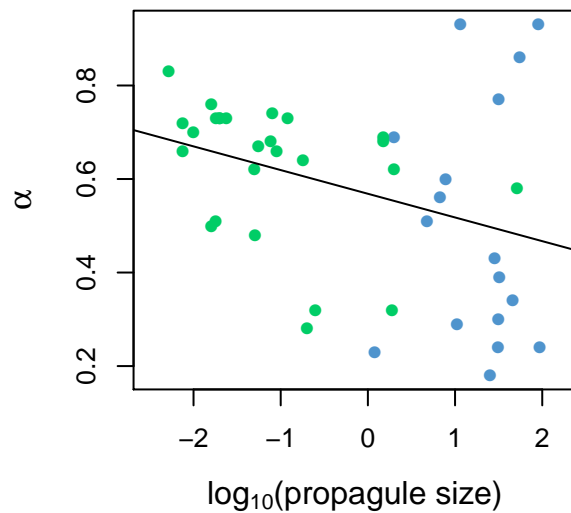

Supplement: S3 Fig — n = 43 species; blue: vertebrates; green: invertebrates; a: r2 = 0.13, p-val = 0.02; b: r2 = 0.12, p-val = 0.02; c: r2 = 0.005, not significant; d: r2 = 0.12, p-val = 0.02. (PDF) [file pgen.1005774.s004.pdf]

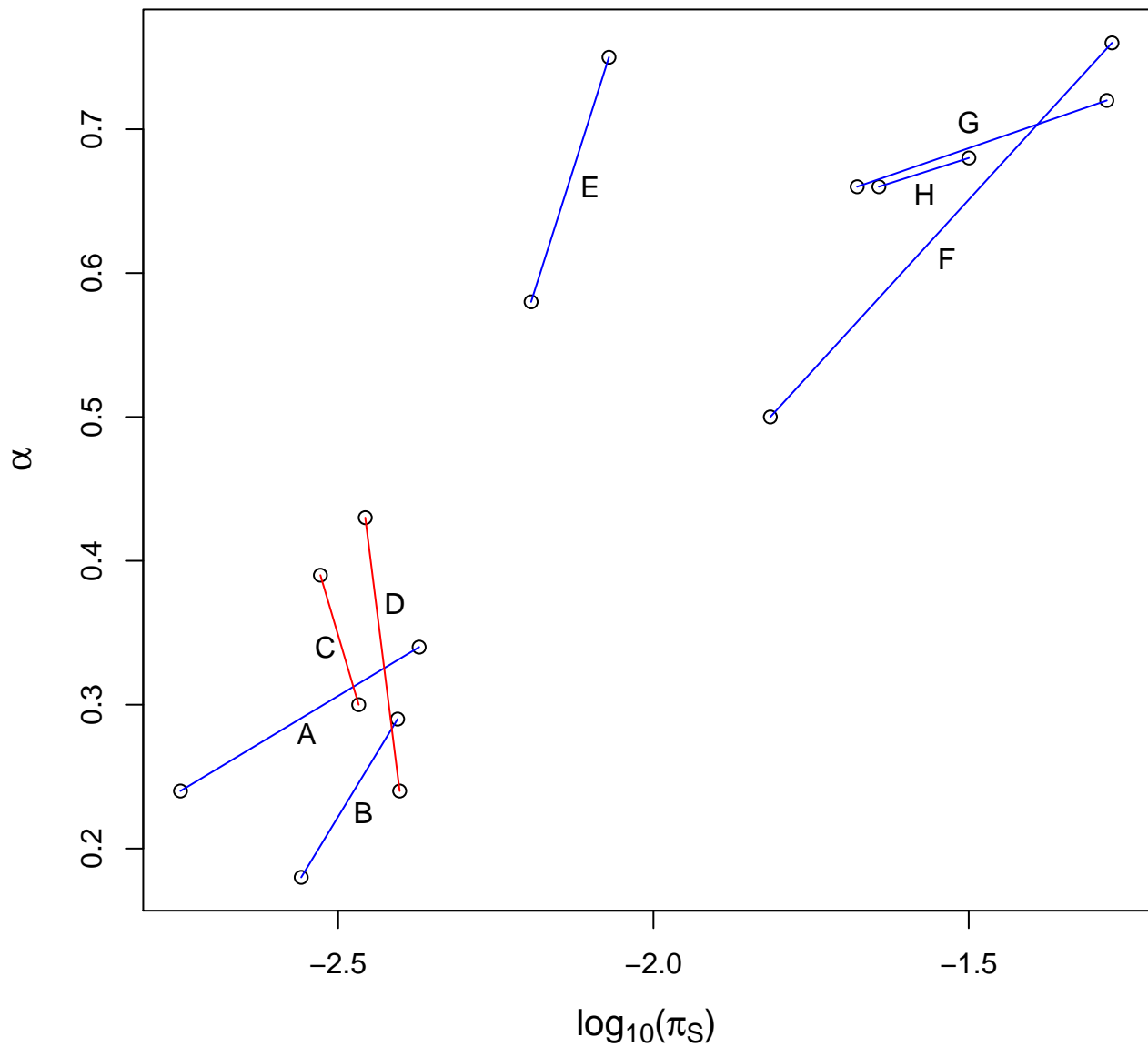

Supplement: S4 Fig — Segments connect pairs of mirror species. Blue: increasing α with πS; red decreasing α with πS; A: (Homo sapiens, Pan troglodytes); B: (Nycticebus coucang, Galago senegalensis); C: (Eulemur mongoz, Eulemur coronatus); D: (Chlorocebus aethiops, Macaca mulatta); E: (Varecia variegata variegata, Propithecus coquereli); F: (Ciona intestinalis A, Ciona intestinalis B); G: (Echinocardium mediterraneum, Echinocardium cordatum B2); H: (Thymelicus sylvestris, Thymelicus lineola) (PDF) [file pgen.1005774.s005.pdf]
