# Supplementary material for: Adaptive Protein Evolution in Animals and the Effective Population Size Hypothesis
Source: PLoS Genet. 2016 Jan 11;12(1):e1005774. doi: 10.1371/journal.pgen.1005774 (PMC4709115; doi:10.1371/journal.pgen.1005774)
Supplement: S1 Text — (PDF) [file pgen.1005774.s006.pdf]

Adaptive Protein Evolution in Animals and the Effective Population Size Hypothesis

Supporting Information: S1 Text – Simulation protocol and results

The reliability of the newly introduced methods was assessed by analysing simulated data sets. Forward simulations were performed using SLIM v1.8 [1] in a way very similar to the Messer and Petrov (2013) study [2].

1. Constant population size

1.1 Simulation protocol

SLIM models mutation, drift, selection and linkage. We simulated the evolution of 250 equally-spaced genes carried by a 10 Mb-long chromosome (one gene every 40 kb). Each gene was made of eight exons of length 150 bp each, separated by introns of length 1.5 kb. Genes were flanked by a 550-bp–long 5′ UTR and a 250-bp–long 3′ UTR. One fourth of the coding and UTR positions were assumed to evolve neutrally, and three fourth were assumed to be under selection. We used a mutation rate of 2.5×10^{-8} per site and generation, a recombination rate of 10^{-8}, and a panmictic diploid population of constant size $N = 10^4$. These are identical to the settings of Messer and Petrov (2013).

The selected mutations were codominant and either deleterious or beneficial, the proportion of beneficial ones being called p. The selection coefficient of advantageous mutations was fixed to s_b=10^{-3}. The selection coefficients of deleterious mutations followed a Gamma distribution of shape 0.3 and mean s_d. p and s_d were varied among simulations in order to obtain a wide range of simulated α while keeping the simulated d_N/d_S similar to that of real data sets.

Simulations were run during 10^6 generations. Every 10^5 generations a sample of 7 diploid individuals was taken and allele frequencies at segregating positions were recorded, separately for neutral and selected mutations (simulated SFS). For each allele frequency category, counts were summed across the ten $k.10^5$th generations. At generation 10^6, substituted positions were counted, separating neutral, deleterious and beneficial mutations, only counting mutations having appeared after generation 10^5 (burn-in). This is equivalent to simulating a set of 2,500 genes carried by ten distinct chromosomes during 10^5 generations. Twelve distinct data sets were generated this way, each using a different (p, s_d) pair. Each simulation took ~1.5 day. Three estimates of α and ω_a were calculated for each simulated data set, namely the Gamma estimate, the GammaExponential estimate, and the ScaledBeta estimates (see main text). They were compared to the "true" (=simulated) values. These methods assume free recombination between loci and SNPs. The simulation model is therefore different from the inference models.

1.2 Results

The number of simulated neutral SNPs was ~3500 per data set, and the number of simulated neutral substitutions ~2800, which is reasonably similar to the real data sets analysed in this study (see S1 Table). The simulated α varied between 0 and 0.63 across data sets, the simulated between 0 and 0.12, and the simulated d_N/d_S ratio between 0.06 and 0.26. The estimated α and ω_a were plotted against the simulated (true) ones:

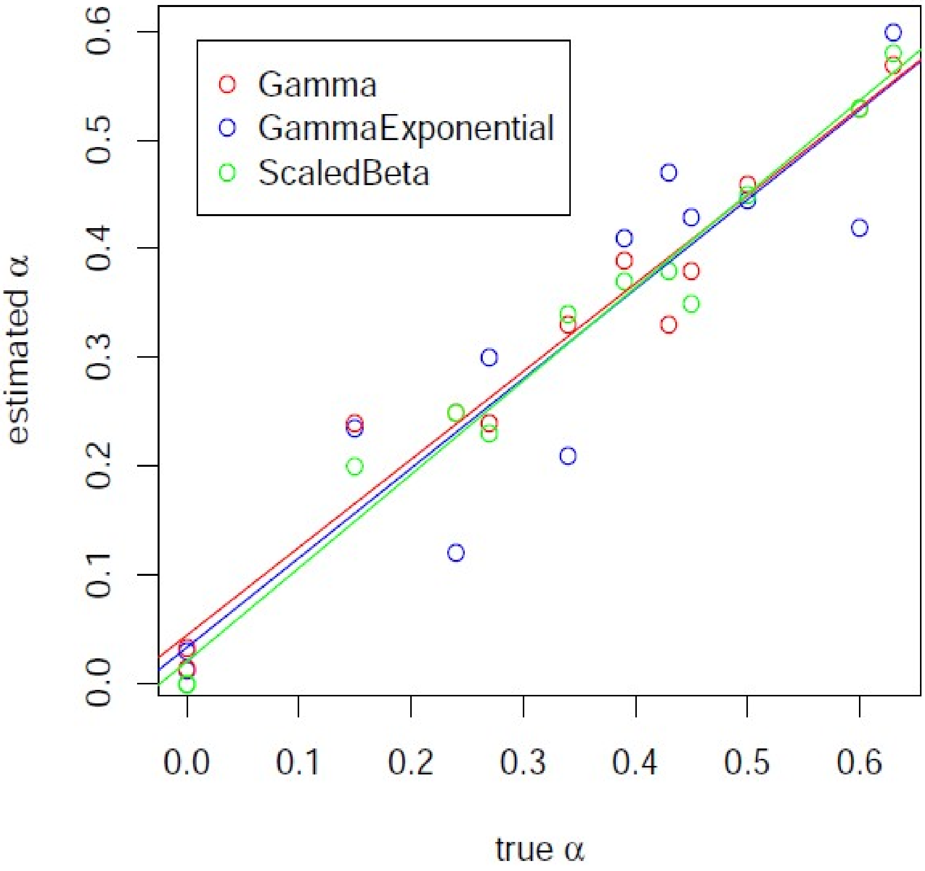
Gamma
GammaExponential
ScaledBeta
estimated α
true α
0.0
0.1
0.2
0.3
0.4
0.5
0.6

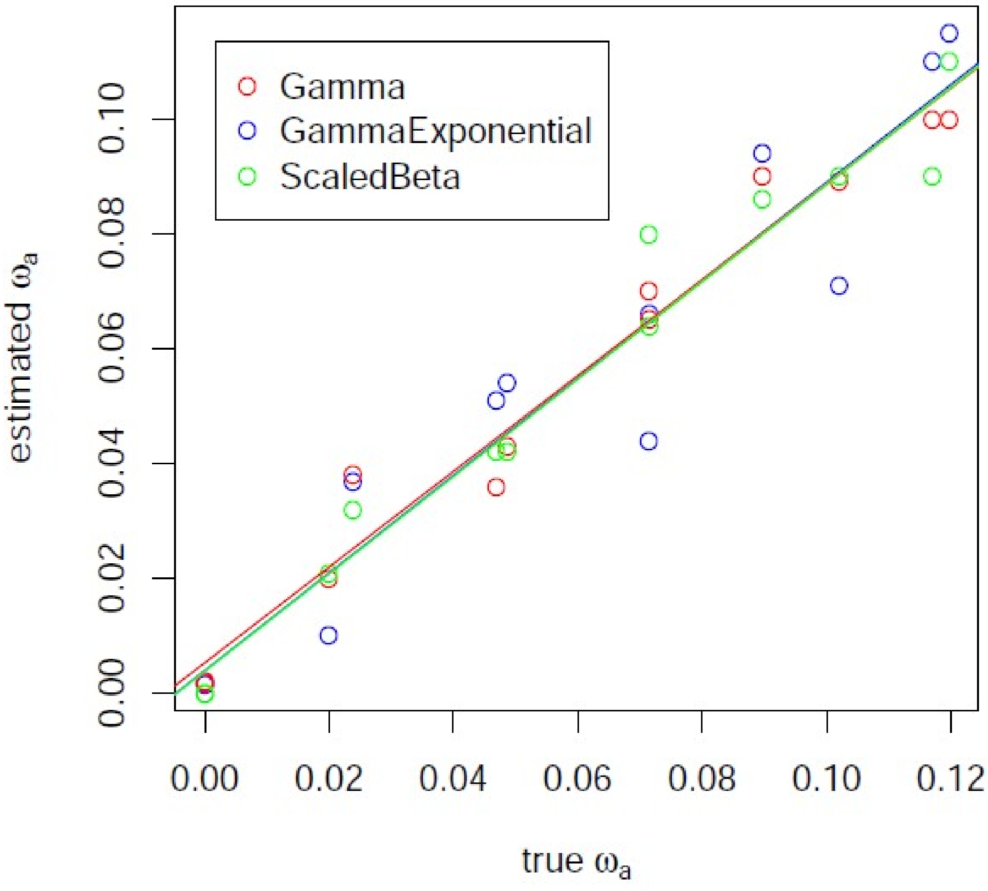
Gamma
GammaExponential
ScaledBeta
estimated ω_a
true ω_a
0.00
0.02
0.04
0.06
0.08
0.10
0.12

The three estimates performed well; regression lines are almost undistinguishable from the (y=x) line (dotted), and the correlation coefficient between true and estimated values was above 0.9 in all three cases. In all 12 data sets the Gamma model outperformed the GammaExponential and ScaledBeta models according to Akaike's Information Criterion, which was expected since the simulations assume a Gamma distribution of selection coefficients. The latter two models, although over-parametrized in this specific case, did perform quite well as far as α and ω_a estimation were concerned.

These results are in agreement with previous simulation studies, which assessed the accuracy of distinct but related estimators of α. Performing simulations very similar to ours, Messer and Petrov (2013) [2] showed that the Keightley & Eyre-Walker (2007) method [3] returns reasonably good estimates of α in spite of the distortion of SFS's due to linked selection – even though demographic parameters can take irrelevant values in such cases. Eyre-Walker and Keightley (2009) [4] compared the Eyre-Walker et al. (2006) [5] and the Keightley & Eyre-Walker (2007) [3] methods based on real data and simulations. They found that the former, which is very similar to the Gamma estimate of this study, performed a bit better than the latter, both being quite robust to departure from the assumption of independence between SNPs. The simulation scheme of Eyre-Walker and Keightley (2009) [4] did not incorporate any adaptive effect.

2. Fluctuating population size

A second round of simulations was conducted assuming varying effective population (N) in time. $N(t)$ was assumed to follow a Markov process, the average waiting time between two events of population size change being 50,000 generations. After each event of N change, the new N was randomly drawn in the [5,000; 50,000] interval, in such a way that $1/N$ was uniformly distributed over [1/50,000; 1/5,000]. The average N was close to 10^4.

The deleterious effect s_d was set to -0.25, and three positive selection regimes were simulated: p=0 (no adaptive mutation), p=0.0015 (medium), p=0.007 (high adaptive rate). Other parameters were identical to section 1 above. Again, despite some additional sampling variance, the true and estimated α and ω_a were well correlated, suggesting that DFE-based McDonald-Kreitman methods are reasonably robust to fluctuations in effective population size (see figures below).

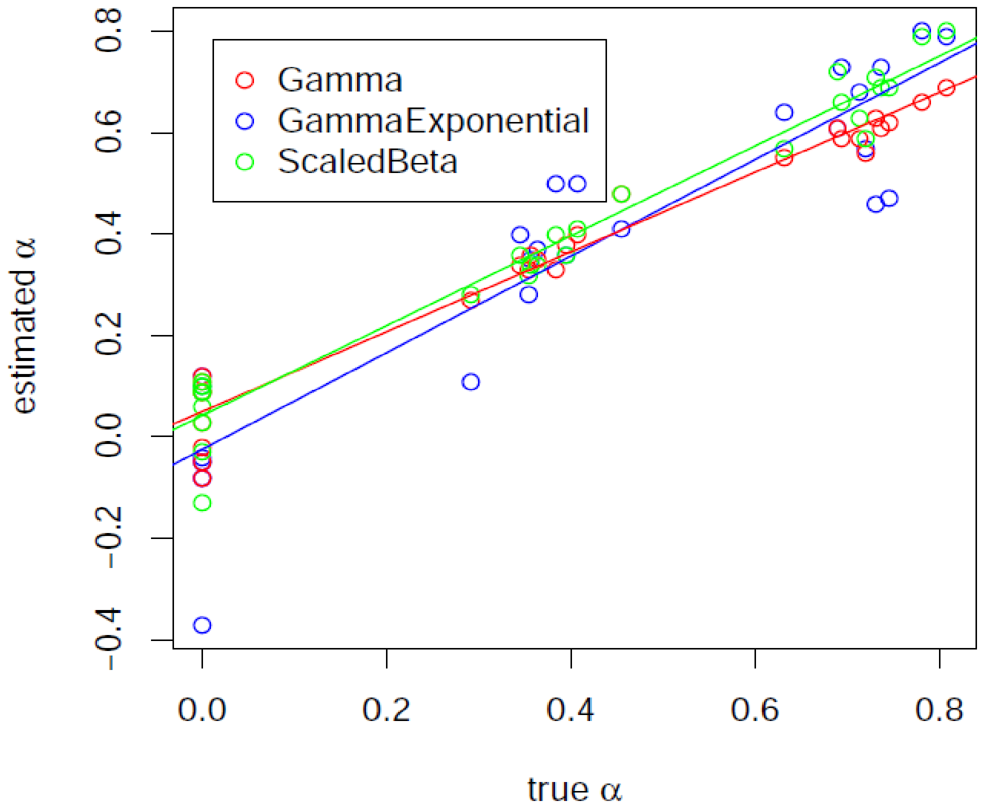

Gamma
GammaExponential
ScaledBeta
estimated α
true α
0.0
0.2
0.4
0.6
0.8
-0.4
-0.2

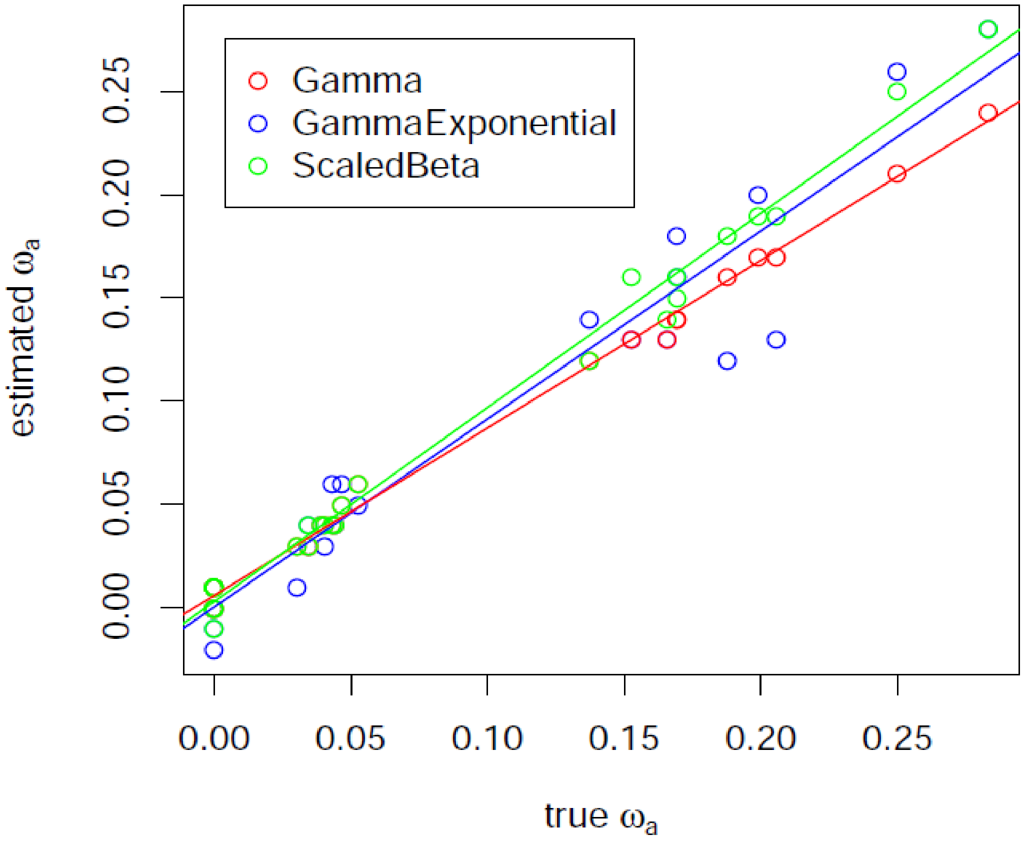

Gamma
GammaExponential
ScaledBeta
estimated ω_a
true ω_a
0.00
0.05
0.10
0.15
0.20
0.25

References

[1] Messer PW. SLiM: simulating evolution with selection and linkage. Genetics. 2013 Aug;194(4):1037-9.

[2] Messer PW, Petrov DA. Frequent adaptation and the McDonald-Kreitman test. Proc Natl Acad Sci U S A. 2013 May 21;110(21):8615-20.

[3] Keightley PD, Eyre-Walker A. Joint inference of the distribution of fitness effects of deleterious mutations and population demography based on nucleotide polymorphism frequencies. Genetics. 2007 Dec;177(4):2251-61.

[4] Eyre-Walker A, Keightley PD. Estimating the rate of adaptive molecular evolution in the presence of slightly deleterious mutations and population size change. Mol Biol Evol. 2009 Sep;26(9):2097-108.

[5] Eyre-Walker A, Woolfit M, Phelps T. The distribution of fitness effects of new deleterious amino acid mutations in humans. Genetics. 2006 Jun;173(2):891-900.
